# Prevalence and risk factors of urinary tract infection in kidney recipients: a meta-analysis study

**DOI:** 10.1186/s12882-023-03338-4

**Published:** 2023-09-27

**Authors:** Masoumeh Hosseinpour, Aiyoub Pezeshgi, Morteza Zaboli Mahdiabadi, Foroogh Sabzghabaei, Hamed Hajishah, Soheila Mahdavynia

**Affiliations:** 1 Faculty of Medical Sciences, Tabriz Branch, Islamic Azad University, Tabriz, Iran; 2 Faculty of Medicine, Zanjan University of Medical Sciences, Zanjan, 83153-45139 IR Iran; 3 Student Research Committee, Shahid Sadoughi University of Medical Sciences, Yazd, Iran; 4 Firoozabadi Clinical Research Development Unit (FACRDU), Iran University of Medical Sciences, Tehran, Iran; 5 Student Research Committee, Tehran Medical Sciences Branch, Islamic Azad University, Tehran, Iran

**Keywords:** Urinary tract infection, Risk factors, Prevalence, Kidney transplant

## Abstract

**Background:**

A kidney recipient's urinary tract infection (UTI) can result in infectious problems and be a risk factor for less successful transplant outcomes. UTI risk factors are still controversial. The present study aimed to investigate the prevalence of UTI and its association with risk factors in kidney recipients.

**Method:**

Twenty-six papers published between 2005 and 2022 were retrieved using keywords and searching Medlib, ScienceDirect, PubMed, and other databases. If possible, the pooled prevalence of UTI in kidney recipients and odds ratio (OR) with a 95% confidence interval for each risk factor were calculated. The data were analyzed using the random effects model in R and Stata 14.

**Results:**

The total sample size was 72,600, with an average age of 48.7 years. The pooled prevalence of UTI was 35% (95% CI, 30–40%). The estimated risk factors for UTI were female (OR = 3.13; 95%CI: 2.35—4.17), older age (OR = 1.03; 95%CI: 1—1.05), history of UTI (OR = 1.31; 95%CI) CI: 1.05—1.63), receiving a kidney from a deceased donor (OR = 1.59; 95%CI: 1.23—2.35), long-term use of an indwelling catheter (OR = 3.03; 95%CI: 1.59—6.59), a ureteral stent (OR = 1.54; 95%CI: 1.16—2.06), diabetes (OR = 1.17; 95%CI: 0.97—1.41), hypertension (OR = 1.6; 95%CI: 1.26—2.28), acute rejection process (OR = 2.22; 95%CI: 1.45—3.4), and abnormal urinary tract anatomy (OR = 2.87; 95%CI 1.44—5.74).

**Conclusion:**

This meta-analysis revealed that UTIs are a significant problem in kidney recipients. Factors such as female sex, old age, history of UTIs, deceased donor, long-term use of an indwelling catheter, diabetes, acute rejection process, use of ureteral stent, abnormal urinary tract anatomy, and hypertension were related to an increased risk of UTIs in kidney recipients.

## Introduction

Kidney transplantation has gained popularity as the preferred medical procedure for the majority of patients in recent years with advanced and chronic kidney failure, which improves their quality of life and increases their life expectancy [1, 2]. Despite progress in this field, post-transplant infections remain a major cause of death in kidney recipients, including active infections preventing transplants and treatable chronic infections before transplantation [3]. Bacterial infections are among the most critical causes of transplant rejection and mortality in the early post-transplantation stages [4–81]. Around 80% of transplant recipients get infections within the first post-transplant year due to factors like potent immunosuppression, surgery, and continuous exposure to hospital-acquired pathogens [4–6]. Infection and dysfunction of the internal organs have a close and strong relationship with using immunosuppressive drugs post-transplant [7]. The most important risk factors that cause post-transplantation infection are the amount and initial dose of immunosuppressive drugs, the manner and degree of continuing immunosuppression during treatment, and the process of acute transplant rejection [8]. Prevention procedures, quick diagnosis, and treatment of this infection are vital [9]. Various studies have shown that UTIs are the most common infection among kidney recipients worldwide [10]. UTIs are one of the main causes of complications and hospitalization after kidney transplantation and seriously threaten successful transplantation outcomes [11, 12]. Almost one out of four kidney recipients will have a UTI within one-year post-transplantation, and these infections can negatively impact transplant outcomes if not well-treated [13, 14]. The prevalence of bacterial infections in different countries varies from 35 to 79%, and about 60% of nosocomial septicemias in kidney recipients are caused by UTIs [15, 16]. UTIs are usually expected in a short period post-transplantation [17]. The risk of getting an infection 3 to 6 months post-transplantation is equal to the general population, and this late infection has a better prognosis than early urinary infection [18, 19]. Since urinary infection in the first three months, post-transplantation is usually asymptomatic, in many cases [81], it can manifest itself with pyelonephritis, bacteremia, dysfunction of the transplanted organ, and a high risk of bacterial infection recurrence [20]. Correct diagnosis of UTI and appropriate treatment play a significant role in preventing transplant rejection and mortality. Risk factors related to the development of UTIs include sex, age, dose and duration of immunosuppression, co-morbidities such as diabetes mellitus (DM), aggressive urological maneuvers, and delay in transplant function as the most important parameters involved [23]. In addition, urinary tract instruments, including urinary catheters and ureteral stents, have also been identified as potential risk factors for UTIs post-transplantation [24]. Kidney recipients' most common pathogens leading to UTIs include *Enterobacteriaceae, Enterococci, Staphylococci,* and* Pseudomonas*. Other less prevalent microorganisms include *Salmonella, Candida,* and* Corynebacterium uroliticum*. Moreover, there is a possibility of infection by uncommon pathogens such as *Mycoplasma hominis*, *Mycobacterium tuberculosis,* or JC and BK viruses [25]. The most common symptoms of lower UTI are frequent urination and urgency due to cystitis. However, symptoms of more severe infection, such as fever, kidney allograft sensitivity, and sepsis due to acute pyelonephritis can also be seen [24]. Considering the importance of timely diagnosis and treatment to prevent life-threatening complications and transplant loss, it is necessary to identify the risk factors of UTI.

Given the importance of kidney transplants, we reviewed the data of different studies to identify the prevalence and influential risk factors for the development and progression of UTI in kidney recipients.

## Method

### Search strategy

This meta-analysis investigated the prevalence and risk factors for UTI in kidney recipients. Electronic documents and resources available until July 2022 were reviewed. Scientific journals and papers in PubMed, Medlib, ScienceDirect, ISI, Scopus, and Embase databases were retrieved. Articles were searched mainly using valid keywords such as kidney transplant, kidney transplantation, renal transplant, organ transplantation, organ transplant, urinary tract infection, UTI, infection, factors, and possible combinations in English. Keywords were standardized in MeSH and used for searching.

### Inclusion and exclusion criteria

The inclusion criteria were: (1) studies that included adult patients receiving kidney transplants; (2) studies that investigated risk factors for UTI in patients after kidney transplantation; (3) studies in which immunosuppressant guidelines were similar after kidney transplantation; (4) the definition and diagnostic criteria of UTI were the same as the criteria of the Centers for Disease Control and Prevention (positive urine culture, i.e., ≥ 105 microorganisms per cc of urine) or clinical manifestations of fever > 38 °C, dysuria, urinary frequency, and urinary urgency, in the absence of pyelonephritis and the and criteria for cystitis).

The exclusion criteria were: (1) studies that included subjects with kidney transplant dysfunction caused by an acute disease other than UTI (e.g., myocardial infarction, acute intra-abdominal disorders, stroke); (2) studies that lacked risk factors for UTI or insufficient data to calculate the odds ratio (OR) of UT; (3) qualitative and descriptive studies; (4) abstract only, conference papers, reviews, systematic reviews, and meta-analyses; (5) studies published in languages other than English.

### Study selection

Using Endnote X8, two researchers examined the titles and abstracts of the papers and then screened them according to the inclusion and exclusion criteria. Articles that met the requirements were further evaluated by reading their full text. In a disagreement between the two researchers, a third researcher passed the final judgment.

The selected documents were thoroughly reviewed, and all their information was entered into a data extraction form; then, the data were imported into Microsoft Excel. In the next step, the data were transferred from Excel to Review Manager 5.3 and Stata 14. The data collected in this study included the author's name, year of publication, location of research, number of patients, mean age, duration of follow-up, design, female/male, deceased donors/living donors, number of UTIs, risk factors of UTI including underlying disease (diabetes, hypertension), use of ureteral stents, days of catheterization, history of UTI, acute rejection process (ACR), abnormal anatomy of the urinary tract, and the abundance of UTI-causing bacteria. The primary objective was to investigate the prevalence of UTI in kidney recipients, and the main goal was to examine the risk factors of UTI in these patients.

### Risk of bias assessment

**Fig. 1:**
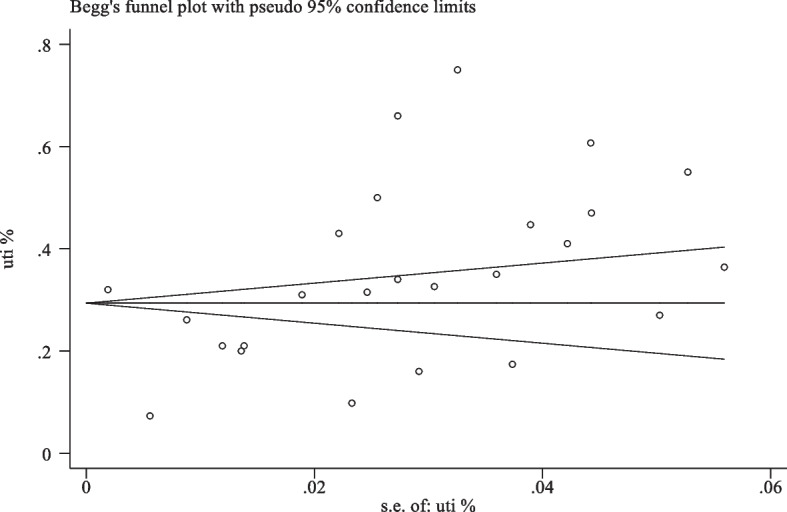
Publication bias test using Begg’s funnel plot test

Two reviewers independently evaluated the study's quality using the Newcastle–Ottawa checklist. Discussions with the third reviewer helped to resolve discrepancies. Scores under 3, under 6, and between 7 and 9 were regarded as low, moderate, and high-risk studies, respectively. The total score varied from 0 to 9. The discussion was used to settle any disagreements between the two investigators in each process. The details of risk of bias assessment are available in Table 1. Also, publication bias was evaluated by visual inspection of funnel plot asymmetry (Fig. 1).

**Table 1:** Risk of bias assessment of the included studies

**Study (Ref)**	**Exposed representation**	**Nonexposed selection**	**Selection Ascertainment of obesity**	**Outcome absent at study start**	**Comparability**	**Outcome assessment**	**Outcome Follow-up length**	**Adequacy of follow-up**	**Overall score**	**Risk of bias**
					**Adjustment by age and nodal status or stage**					
Bonkat G [42]	**1**	**1**	**-**	**1**	**1**	**1**	**1**	**1**	**7**	Low
Alangaden G J [5]	**-**	**1**	**1**	**1**	**1**	**1**	**1**	**1**	**7**	Low
Sorto R [43]	**1**	**1**	**-**	**1**	**1**	**1**	**1**	**1**	**7**	Low
Golebiewska J [69]	**1**	**1**	**1**	**1**	**1**	**1**	**1**	**1**	**8**	Low
Farr A [70]	**1**	**1**	**-**	**1**	**1**	**1**	**1**	**1**	**7**	Low
Papasotiriou M [23]	**1**	**1**	**1**	**1**	**1**	**1**	**1**	**1**	**8**	Low
Vidal E [33]	**1**	**1**	**1**	**1**	**1**	**1**	**1**	**1**	**8**	Low
Giullian JA [71]	**1**	**1**	**-**	**1**	**-**	**1**	**1**	**1**	**6**	Moderate
Dantas S [39]	**1**	**-**	**1**	**1**	**-**	**1**	**1**	**1**	**6**	Moderate
Chuang P [5]	**1**	**1**	**1**	**1**	**1**	**1**	**1**	**1**	**8**	Low
Pellè G [72]	**1**	**1**	**1**	**1**	**1**	**1**	**1**	**1**	**8**	Low
Memikoglu KO [73]	**1**	**1**	**1**	**1**	**1**	**1**	**1**	**1**	**8**	Low
ópez-Medrano F [74]	**1**	**1**	**1**	**1**	**1**	**1**	**1**	**1**	**8**	Low
Safdar N [44]	**1**	**-**	**1**	**1**	**1**	**1**	**1**	**1**	**7**	Low
Wojciechowski D [10]	**1**	**1**	**-**	**1**	**-**	**1**	**1**	**1**	**6**	Moderate
Lee JR [12]	**1**	**1**	**1**	**1**	**1**	**1**	**1**	**1**	**8**	Low
Espinar MJ [61]	**-**	**1**	**-**	**1**	**1**	**1**	**1**	**1**	**6**	Moderate
Bodro M [75]	**1**	**1**	**1**	**1**	**1**	**1**	**1**	**1**	**8**	Low
Królicki T [76]	**1**	**1**	**-**	**1**	**1**	**1**	**1**	**1**	**7**	Low
Bodro M [45]	**1**	**1**	**1**	**1**	**1**	**1**	**1**	**1**	**8**	Low
Britt NS [62]	**1**	**1**	**1**	**1**	**1**	**1**	**1**	**1**	**8**	Low
Ariza-Heredia EJ [67]	**1**	**1**	**1**	**1**	**1**	**1**	**1**	**1**	**8**	Low
Naik AS [77]	**1**	**1**	**1**	**1**	**1**	**1**	**1**	**1**	**8**	Low
Liu S [78]	**-**	**1**	**1**	**1**	**1**	**1**	**1**	**1**	**7**	Low
Hazratullah E [63]	**_**	**1**	**1**	**1**	**_**	**1**	**-**	**1**	**5**	Moderate
Mansury D [79]	**1**	**1**	**1**	**1**	**_**	**1**	**1**	**1**	**7**	Low

### Statistical analysis

The pooled prevalence of UTI in kidney recipients was calculated with a 95% confidence interval, and subgroup analysis was performed according to the geographical area of the studies and the type of antibiotic prophylaxis. The odds ratio (OR) with a 95% confidence interval for each risk factor was recorded or calculated when possible. Studies were pooled according to the sample size, mean, and standard deviation. Each study was weighted according to the inverse of its variance. The Q test and I^2^ index were tested for significance at the α error level of < 10% to investigate heterogeneity. In cases where the results of the studies were heterogeneous, they were analyzed using meta-analysis (random effects model). R and Stata 14 were used for data analysis. A random effects model calculated ORs and the corresponding 95% confidence intervals (CIs) for dichotomous outcomes.

## Results

**Fig. 2:**
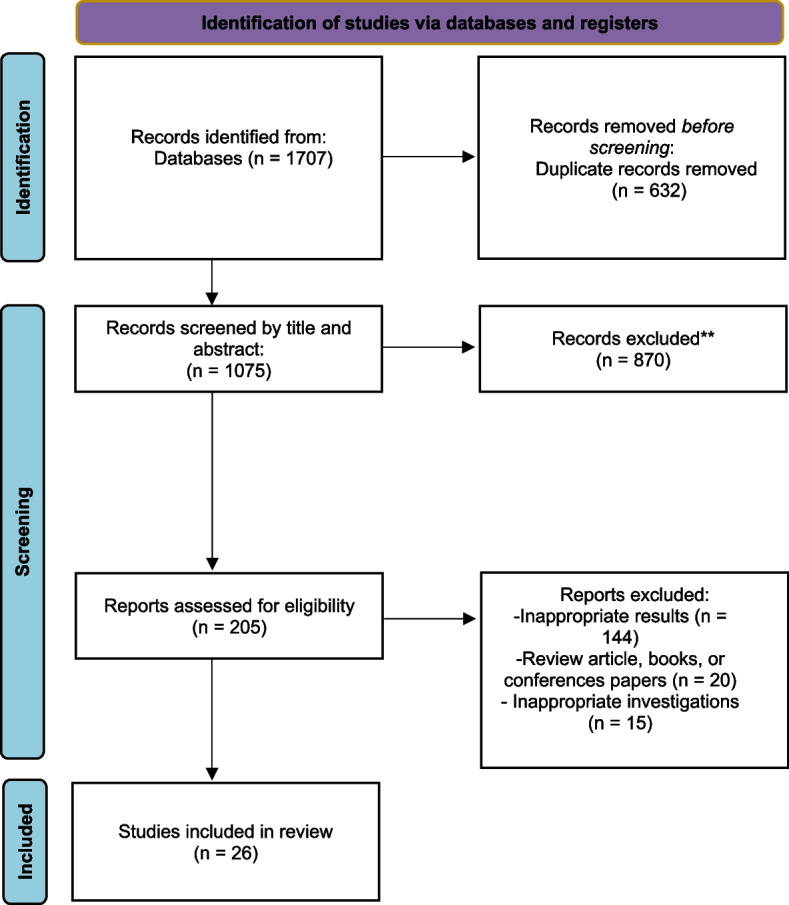
Flow diagram of studies identified in this study

After removing duplicates and irrelevant studies, 205 studies were examined. The steps of study selection are shown in Fig. 2. Finally, 26 eligible papers published between 2005 and 2022 were included in this meta-analysis (Table 2) (Fig. 1). These studies were conducted on 72,600 participants, with an average age of 48.7 years. Moreover, 59.8% of the participants were men, and 40.2% were women. The follow-up periods in the included studies varied considerably, with the longest extended study period being 11 years and the shortest being three months.

**Table 2:** Characteristics of the articles reviewed in this study

**AUTHOR Ref**	**Year**	**No. of participants**	**Country**	**Mean age**	**Male/female (n)**	**% UTI**	**Deceased donor/ living donor**	**Antibiotic prophylaxis**	**Study design**	**Risk factors investigated**	**Mean follow-up (mo)**
Bonkat G [42]	2012	78	Switzerland	56	51/27	27	50/28	Sulfanamid	Cohort	Age, female sex, previous history of UTI	24
Sorto R [43]	2010	176	Mexico	37	96/80	35	38/138	B lactam	Cohort	Age, female sex, deceased donor, duration of catheter, diabetes, use of ureteral stent, abnormal anatomy of urinary tract, antibiotic prophylaxis	48
Golebiewska J [69]	2011	89	Poland	48.1	52/37	55	88/1	B lactam	Cohort	Age, female gender, previous history of UTI, acute rejection, use of ureteral stent	12
Farr A [70]	2014	598	Austria	54	389/209	31	57/521	Sulfanamid	Cohort	Age, female gender	18
Papasotiriou M [24]	2011	122	Greece	44	75/47	60.7		Sulfanamid	Cohort	Female gender, diabetes, acute rejection	68
Vidal E [33]	2012	2172	Spain	52	1381/671	7.3		Sulfanamid	Cohort	Age, female sex, previous history of UTI, diabetes, acute rejection	18
Giullian JA [71]	2008	158	USA	47	109/49	16	67/76	Sulfanamid	Cohort	Female gender, deceased donor, Diabetes, abnormal anatomy of the urinary tract, antibiotic prophylaxis	36
Dantas S [39]	2006	163	Brazil	42.5	98/65	44.7	110/53	Sulfanamid	Cohort	Deceased donor, duration of catheter	24
Chuang P [6]	2005	500	USA	44	331/169	43	105/195	B lactam	Cohort	Age, female gender, deceased donor	42
Pellè G [72]	2007	177	France	46.5	117/60	75	153/24	B lactam	Cohort	Female gender, previous history of UTI, acute rejection,	22
Memikoglu KO [73]	2007	136	Turkey	32	88/48	41	33/103	B lactam	Cohort	Female gender, use of ureteral stents	38
ópez-Medrano F [74]	2014	163	Spain	44.8	107/56	9.8	140/244		Cohort	Female gender	26
Safdar N [44]	2005	385	USA	47	166/218	50	607/141		Cohort	Female gender, previous history of UTI, duration of catheter, diabetes, use of ureteral stent, abnormal anatomy of urinary tract, antibiotic prophylaxis	96
Wojciechowski D [11]	2013	236	USA	51.6	145/91	32.6	95/141	Sulfanamid	Cohort	Age, female gender, deceased donor, use of ureteral stent	12
Espinar MJ [61]	2015	98	Portugal	53.8	35/63				case control	Previous history of UTI, diabetes, antibiotic prophylaxis	9
Bodro M [75]	2015	867	Spain	60		20		Sulfanamid	Cohort	Age, female gender	90
Bodro M [45]	2015	867	Spain	60	520/347	21	662/205	Sulfanamid	Cohort	Female gender, deceased donor, diabetes, acute rejection	90
Ariza-Heredia EJ [67]	2014	301	USA	56.7	177/124	34	123/132	B lactam	Cohort	Female gender, use of ureteral stents, abnormal anatomy of the urinary tract	24
Naik AS [77]	2015	60,702	USA	52	36,725/23977	32	44,616/16086		Cohort	Age, female gender, deceased donor, diabetes	132
Liu S [78]	2016	103	China	35.4	79/24	17.4		B lactam	RCT	Age, female sex, use of ureteral stent	48
Hazratullah E [63]	2022	74	Pakistan		62/12	36.4		Sulfanamid	Cohort	Female gender, previous history of UTI, diabetes, acute rejection, abnormal anatomy of the urinary tract	3
Hazratullah E [63]	2022	74	Pakistan		62/12	36.4		Sulfanamid	Cohort		
Mansury D [79]	2018	356	Iran		206/150	31.5	241/115		Cohort	Female gender, deceased donor,	48

### Prevalence of UTIs

**Fig. 3:**
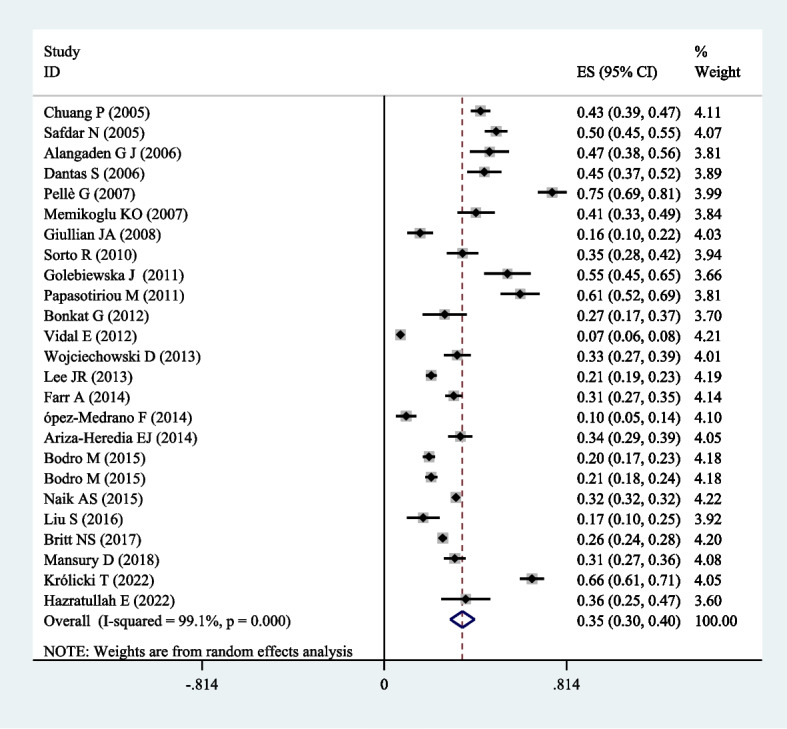
Forest plot of the prevalence of UTIs in renal transplant patients. The square represents the effect estimate of individual studies with their 95% confidence intervals with the size of squares proportional to the weight assigned to the study in the meta-analysis. In this chart, studies are stored in order of the year of publication and author’s names, based on a random effects model

The pooled prevalence of UTIs was 35% (95% CI, 30%-40%; *P* < 0.01) in the entire population (Fig. 3). The prevalence of UTIs varied in the reviewed studies. The lowest prevalence of UTIs was 7.3%, and the highest prevalence was 75%. In the subgroup analysis based on the studied geographical region, the prevalence of UTIs was 34% in America based on 11 studies, 37% in Europe based on ten studies, and 31% in Asia based on four studies. Moreover, in the subgroup analysis based on antibiotic prophylaxis, the prevalence of UTIs was 41% in patients who received beta-lactam after kidney transplantation based on nine papers, and 29% in patients who received sulfonamide based on 11 articles (Table 3).

**Table 3:** The prevalence of UTIs in kidney transplant patients based on subgroup analysis of location and type of antibiotic

	**Subgroup**	**Number of articles**	**UTI (% (95% CI)**
	**America**	11	34% (30% to 38%)
**Location**	**Europe**	10	37% (25% to 39%)
	**Asia**	4	31% (22% to 41%)
**Antibiotic**	**β-lactam**	9	41% (29% to 53%)
	**Sulfonamide**	11	29% (21% to 36%)

### Risk factors for UTIs in patients after kidney transplant

Twelve papers examined the association between the recipient's age and the occurrence of UTIs. The pooled OR for older age was 1.03 *P* = 0.11). The relationship between sex and UTIs was investigated in 23 papers. As shown in Table 4, the incidence of UTIs in women was significantly higher. The pooled OR for the female sex was 3.13 (*P* < 0.001). The relationship between a history of UTIs and UTIs post-transplant was discussed in eight papers. The pooled OR of a history of UTIs pre-transplantation was 1.31 ( *P* = 0.001). Pooled results from 11 papers demonstrated that the incidence of UTIs in patients who received a kidney from a deceased donor was 1.59 (*P* < 0.001) times higher than that of those who received a kidney from a living donor.

**Table 4:** The pooled odds ratio for UTIs risk factors in kidney transplant patients

Variable	Number of articles	Pooled odds ratio	*P* value
		**95% Confidence interval (%)**	
Age	12	1.03 (1 to 1.05)	0.11
Female sex	23	3.13 (2.35 to 4.17)	0
previous UTI	8	1.31 (1.05 to 1.63)	0.001
Deceased donor	11	1.59 (1.23 to 2.35)	0
Duration of catheter	4	3.03 (1.59 to 6.59)	0
Diabetes	12	1.17 (0.97 to 1.41)	0
Acute rejection	7	2.22 (1.45 to 3.4)	0
Ureteral stent	10	1.54 (1.16 to 2.06)	0
Abnormal urinary	5	2.87 (1.44 to 5.74)	0
anatomy			
Hypertension	3	1.6 (1.26 to 2.28)	0.12
Antibiotic prophylaxis	6	1.67 (1.34 to 2.37)	0.487

By comparing the data about the duration of catheterization and urinary infections from 4 papers, we concluded that the risk of developing a urinary infection in patients with a longer period of catheter use is higher; pooled OR was 3.03 (*P* < 0.001). Twelve papers investigated the association between diabetes and the occurrence of UTIs. Pooled OR results showed that the odds of developing a UTI in patients with diabetes compared to non-diabetic patients was 1.17 (*P* < 0.001). The association between aacute rejection process and UTI was investigated in 7 papers. The pooled OR was 2.22 (*P* < 0.001). We pooled data on ureteral stent use and UTIs from 10 papers and concluded that patients who used ureteral stents were more susceptible to UTIs; pooled OR was 1.45 (*P* < 0.001).

Five studies investigated the relationship between UTIs and abnormal anatomy of the urinary tract; a pooled OR of 2.87 (*P* < 0.001) was obtained. Three studies investigated the relationship between UTI and hypertension. The pooled OR for hypertension was 1.6 (*P* = 0.1^2^). More statistical details are noted in Table 4.

**Fig. 4:**
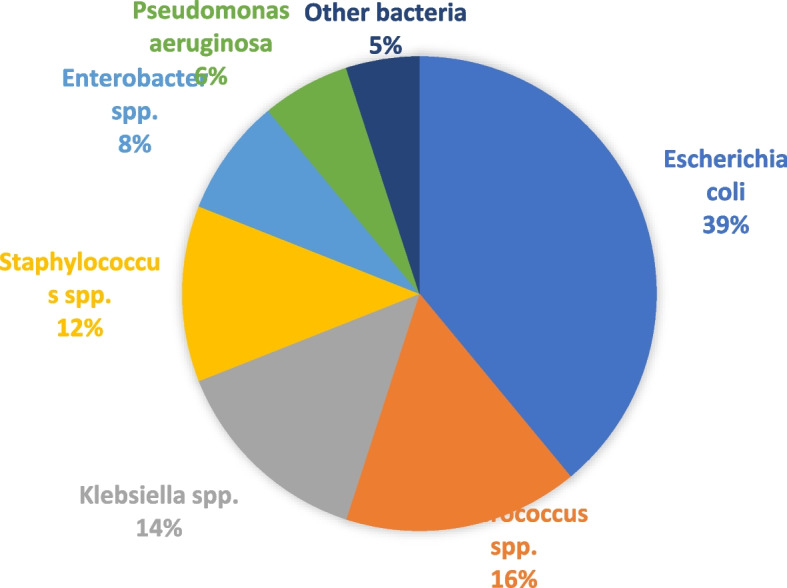
The most important bacteria causing UTIs in kidney transplant recipients

Microbiology of UTIs:

The most common pathogens reported by urine culture in kidney recipients were *Escherichia coli* (39%), and *Enterococcus spp*. (16%), *Klebsiella spp.* (14%), *Staphylococci spp*. (12%), *Enterobacter cloacae* (8%), and *Pseudomonas aeruginosa* (6%) (Fig. 4).

## Discussion

UTIs are prevalent and serious infections after kidney transplantation [6, 26–28]. It has been reported that UTIs can be associated with increased complications and mortality [26, 6, 29] and, possibly, a decline in long-term transplant survival [5]. Studies have shown that the incidence of UTIs in kidney recipients is much higher than the rate observed in the general population [30]. Therefore, it is critical to treat and prevent UTIs in kidney recipients.

The present meta-analysis was conducted to identify and pool the findings of previous studies and meta-analyses investigating the prevalence and risk factors for UTIs in kidney recipients.

This meta-analysis collected data from 26 papers related to 72,600 kidney transplant recipients, in which risk factors for UTIs post-transplantation were examined. Due to the significant heterogeneity among studies, the random effects model was used in all stages. A key factor causing heterogeneity in the results of studies is the difference in procedures followed by different studies; the difference in follow-up time, the definition of UTI, and healthcare systems are among the factors that cause this heterogeneity.

Our study showed an overall prevalence of 35% for UTI in kidney recipients. Older age of the transplant recipient, female sex, previous history of UTI, deceased donor, prolonged use of a catheter, diabetes,acute rejection process, use of ureteral stent, abnormal urinary tract anatomy, and hypertension were associated with an increased risk of infection. *E. coli* was the most common bacterium causing UTI in patients after kidney transplants.

According to the studies conducted in different countries, the prevalence of UTI in patients after kidney transplant varies from 6 to 86% [24]. The explanation for this difference depends on several factors, including differences in surgical technique, the definition of UTI, immunosuppressive drugs, and treatment to prevent infection. Our study obtained an overall prevalence of 35% (30%-40%). In the meta-analysis by Wu et al., this rate was 38% [31]. Lo´pez-Medrano et al. [32] and Vidal et al. [33] reported a prevalence of less than 10% for UTIs. In contrast, Pellè et al. [34] and Papasotiriou et al. [24] showed a prevalence of about 70% for UTIs in kidney recipients. Factors including exposure to an intense immunosuppressive regimen, surgical trauma, long-term urinary tract catheterization, ureteral stents, and prolonged hospitalization can explain the higher percentage of infectious complications in these patients compared to the general population [4, 6].

Based on subgroup analysis, there was no significant difference in the prevalence of UTIs between Americans, Europeans (34% vs. 37%), and Americans and Asians (34% vs. 31%). Still, there was a significant difference between Europeans and Asians (37% vs. 31%). This difference can be caused by genetic differences and different individual characteristics in other countries of two continents. In the subgroup analysis based on antibiotic prophylaxis, the prevalence of UTIs in patients who used beta-lactam antibiotics was significantly higher than in those who used sulfonamide antibiotics (41% vs. 29%). The increase of antibiotic resistance among bacteria, including beta-lactamase-producing strains could cause this difference. A type of resistance occurs through the production of beta-lactamases and induces resistance to beta-lactam drugs. These bacteria have recently been discussed as emerging health problems worldwide [35, 36].

UTI is a common infection among both sexes, but due to physiological reasons, it is more common among women [37]. Several studies have shown that the female sex is a risk factor for UTIs after kidney transplantation [5, 6, 13, 38]. However, other studies have not found a significant relationship between increased urinary infection and the female sex [39, 40]. In the present study, a significant relationship between increased UTIs and the female sex was observed. Since UTIs and bacteriuria are generally more common in women than men due to the shorter urethra and the proximity of the urethral opening to the vagina and anus, this observation is justifiable.

Different studies have reported conflicting results about the relationship between recipients' age and the occurrence of UTIs. Some studies reported no association between the age of recipients and the occurrence of UTI [23, 39, 41]. In contrast, others have reported the recipients' old age as a risk factor for UTIs post-transplantation [33, 42, 43]. Our study showed that age is a weak risk factor in kidney transplant recipients. Factors such as increased incidence of benign prostatic hypertrophy, bladder atrophy, impaired mobility, impaired immune system, and poor personal hygiene are among the reasons for the higher risk of UTIs in the elderly compared to the young [24].

We found that using invasive devices such as urinary catheters and other stents was associated with an increased risk of UTI. This finding is consistent with several studies conducted in this field [5, 42–46], although some studies have presented different results [38, 47–49]. Since indwelling catheters and stents are placed during an invasive procedure, it can damage the urinary tract and, as a result, increase the possibility of urinary infection. Moreover, the urinary tract surgical process and technical errors that contaminate the catheter can increase the risk of infection [50]. Therefore, shortening the time or avoiding urethral catheterization, regular urine culture, and early diagnosis of UTIs are required to reduce the incidence of UTIs.

Conflicting results have been reported regarding acute rejection episodes and the occurrence of UTIs. Some studies have reported no association between acute rejection episodes and UTI incidence [23, 46, 51]. On the other hand, several studies have confirmed the relationship between the period of acute rejection and UTIs [52–54]. Similarly, our findings showed that the rate of UTIs rises in patients who had passed an acute rejection period. The treatment of acute rejection requires more intense immunosuppression, which leads to an increased risk of infectious complications post-transplantation; 60% of patients experience at least one infection during the first-year post-transplantation [55]. On the other hand, immunosuppressive treatments lead to the host's weak inflammatory response against bacteria and increase the risk of infection-related complications. When bacteria invade the urinary tract, certain cytokines, including tumor necrosis factor and interleukin 1, 6, and 8, are activated both locally and systemically [52].

Our results showed that UTIs were more common in kidney recipients from deceased donors than living donors. Several studies have reported similar results [52, 56, 57]. According to one study, the prevalence of UTI in patients who received a transplant from a deceased donor and those who received a kidney from a living donor was 70% and 28%, respectively [58]. On the other hand, in a study, a higher prevalence of UTI was reported in recipients of kidneys from living donors [41]. One of the reasons why UTI is more common in recipients of kidneys from deceased donors can be an asymptomatic infection in the kidney donor, and the occurrence of these infections due to the use of immunosuppressive drugs in the recipients while living donors are tested for disease before donation [30, 59]. Furthermore, patients receiving kidneys from deceased donors probably needed more immunosuppressive treatment compared to those receiving kidneys from living donors [32].

Underlying diseases such as diabetes and hypertension are risk factors for UTI in patients after kidney transplants [65]. Kidney recipients with diabetes are more exposed to bacterial and fungal infections [66]. In our study, diabetes and hypertension were associated with increased UTIs risk in kidney recipients. Diabetes can affect the anatomical and functional features of the urinary system and lead to abnormalities in this system. Such abnormalities are increased following the use of medical devices such as urinary catheters and, in turn, extend the infection [37]. Therefore, regular urine cultures and early diagnosis of urinary infections in these patients are necessary.

Our results showed that anatomical abnormalities of the urinary tract are one of the risk factors for UTIs in kidney recipients. Several studies have reported similar findings [6, 46]. In the study by Ariza-Heredia et al., the most common abnormalities leading to UTIs were benign prostatic hypertrophy, ureteral obstruction, bladder dysfunction, urinary incontinence, and vesicoureteral reflux, respectively [67].

In individuals with and without kidney transplants, the microorganisms that cause UTIs are similar. Over 70% of UTIs are caused by infections with gram-negative bacteria [42]. According to various research, *E. coli* has a frequency ranging from 21 to 73% in the general population and kidney transplants, making it the most prevalent UTI pathogen [24]. *Pseudomonas*, *Staphylococcus*, and *Enterobacter* species are the most common agents causing UTI within 3 to 5 weeks after transplantation, while *E. coli* and *Enterococcus* species mainly cause infection within 6 to 12 weeks post-transplantation [68]. In the present study, *E. coli* was the most common cause of UTI after kidney transplant in patients, followed by *Enterococcus, Klebsiella, Staphylococcus,* and* Enterobacter* species.

There are several limitations to this meta-analysis. The first limitation was the criteria based on which UTIs diagnosis is made. The diagnosis of UTIs is mainly based on positive urine culture, and some doctors argue that these patients only have bacteriuria and do not necessarily have UTIs. Second, despite our efforts to present all relevant variables, there is a possibility that not all UTIs risk factors were included in the results due to the presence of diverse variables and the limitations in the original data. For instance, although we aimed to pool the odds ratios for antibiotic prophylaxis as a risk factor, the wide array of variables prevented us from doing so. Third, there is always a risk of publication bias; the quantity of included papers and variations in sample sizes may had an impact on publication bias.

## Conclusion

Kidney transplantation is a complex and important surgical procedure. Post-operative care, follow-up, and diagnosis and management of possible post-transplantation problems are of great significance. Treatment of infection as one of the most essential postoperative complications can reduce the mortality, complications, and costs imposed on patients. This meta-analysis revealed that UTIs are a significant problem in kidney recipients. Factors such as female sex, old age, history of UTIs, deceased donor, long-term use of an indwelling catheter, diabetes, acute rejection process, use of ureteral stent, abnormal urinary tract anatomy, and hypertensionwere related to an increased risk of UTIs in kidney recipients. To investigate the factors affecting UTIs in kidney recipients and to assess the impact of more recent immunosuppressive medications and prophylactic/therapeutic antimicrobial agents on the pattern of post-transplant infectious complications, prospective trials with a large sample size and a longer follow-up period would be beneficial.

## Data Availability

All data generated or analysed during this study are included in this published article.
